# Transient epileptic amnesia: temporal progression of partially treated disease—a case report

**DOI:** 10.1186/s13256-025-05118-0

**Published:** 2025-10-14

**Authors:** Chamila Mettananda, Sachith Mettananda, Kamal Gunarathne, Manjula Caldera

**Affiliations:** 1 Department of Pharmacology, Faculty of Medicine, University of Kelaniya, Ragama, Sri Lanka; 2 Epilepsy Unit, The National Hospital of Sri Lanka, Colombo, Sri Lanka; 3 Neurology Unit, Teaching Hospital, Anuradhapura, Sri Lanka

**Keywords:** Amnesia, TEA, Transient epileptic amnesia, Transient global amnesia, TGA, Transient memory loss, Temporal lobe epilepsy, Case report

## Abstract

**Background:**

Transient memory loss with preserved consciousness needs precise diagnosis, as it could be owing to different causes requiring different management approaches. Differentiation between causes is difficult on first presentation, but it is important, as different causes have different management approaches and can have serious implications on a patient’s life, especially in regards to driving. Transient epileptic amnesia is a treatable condition if diagnosed correctly but can have major consequences when not treated. Transient epileptic amnesia is reported in literature, but reports on the temporal progression of partially treated transient epileptic amnesia are sparse; however, this knowledge could help someone diagnose the disease at least by the second encounter.

**Case presentation:**

We report on a previously healthy, independent, right-handed 72-year-old Sinhalese Sri Lankan man, who had experienced five to seven brief periods of episodic memory loss since 2018, which were attributed to age, anxiety, and stress. He was involved in a car accident in 2000 and remained conscious but had retrograde amnesia. An extensive investigation conducted during his evaluation did not reveal a secondary cause for the accident. He later presented in 2022 with repeated generalized tonic–clonic seizures without secondary cause and an electroencephalogram showed epileptiform activity involving the left temporal lobe during the postictal period. He was diagnosed with transient epileptic amnesia and was started on carbamazepine. His seizures and amnestic episodes settled with the initiation of treatment, and now he is seizure-free after 6 years from the initial presentation of amnestic episodes. However, he has a mild degree of persistent interictal memory impairment.

**Conclusion:**

Transient epileptic amnesia is difficult to diagnose on the first presentation, as it mimics several conditions where there is nonspecific memory loss, and there are often no involuntary movements. However, recurrent and brief amnestic episodes should lead to suspect transient epileptic amnesia over other causes. Transient epileptic amnesia requires a positive diagnosis, as it is treatable if diagnosed. A contraindication to driving and consequences of untreated disease could cause serious consequences, posing a risk to life. This case shows the temporal progression of the disease in a patient with partially treated transient epileptic amnesia.

## Background

Transient amnesia is a common complaint of the elderly and can occur owing to several reasons. It is often ignored unless it presents with some hard evidence of memory loss. Even when an individual presents with a clear episode of memory loss witnessed by a second person, the exact diagnosis of the disease is difficult to confirm on first presentation, as there is often no diagnostic test, and investigations such as routine electroencephalograms (EEG) could be normal and nondiagnostic. The are several causes of transient amnesia, for example, transient global amnesia (TGA), transient epileptic amnesia (TEA), transient ischemic attack (TIA), or psychological amnesia. Amnesia could also be a feature of a migraine aura, the postictal phase, or a metabolic disturbance [1]. Amnesia can be associated with acute metabolic encephalopathies such as hypoglycemia, hyponatremia, and hypoxia, but those will present with other symptoms, such as a reduced level of consciousness, drowsiness, and sweating, resembling autonomic symptoms, but not solely with amnesia, and are unlikely to recur with stereotypic presentations [2, 3]. Transient memory loss with preserved consciousness needs precise diagnosis, as it has implications for the patient’s life but can be treated if diagnosed correctly. Differentiation between TGA and TEA is difficult, but it is important, as management differs. The main presentation of TGA is the sudden but temporary loss of short-term memory and not being able to form new memories. Confusion generally lasts up to 24 hours. This is most commonly seen in patients older than 50 years, and clinically, they have time disorientation and often ask repeated questions regarding the day’s events. The amnestic spells of TGA last for several hours and are associated with typical behavioral features [4, 5]. TGA is not rare and is a benign condition, with most people having only one episode during their lifetime [4]. TEA occurs typically when waking up from sleep and lasts for a short period; the patient cannot remember the amnestic episode and may also have retrograde amnesia [6–8]. TEA is rare but is a serious diagnosis, as seizures could lead to life-threatening accidents, cause interictal memory impairment, and be a contraindication to driving, leading to a serious impact on patients’ independence and daily activities [1]. Recurrent episodes of transient memory loss should raise suspicions of TEA, as TGA usually does not recur. Transient ischemic attack (TIA) is defined as a transient episode of neurological dysfunction caused by focal brain, spinal cord, or retinal ischemia without acute infarction [9]. Amnesia, as the presenting complaint of a TIA, is rare compared with other common presentations of transient ischemic attacks [10, 11]. Only about 0.2% of all patients with TIA or acute ischemic strokes present with amnesia [12]. TIA is associated with metabolic diseases and management is different to other amnesias. Recurrent episodes of transient memory loss should raise suspicions of TEA, as TGA and TIA usually do not recur.

Partially treated TEA, without any ictal activity, later progressing to generalized tonic–clonic seizures over time, has not been well reported in literature. We report the temporal progression of the disease in such a case.

## Case presentation

A previously healthy, independent, right-handed 72-year-old high-functioning Sinhalese Sri Lankan teetotaler man presented following a low-impact car accident. He did not have evidence of any physical injury, including any head injury or impact. He had driven into a wall on the side of the road and was found by bystanders. He was conscious, talking but unable to identify where he was and what happened for about 15–30 minutes following the event, until he became fully oriented after about 30 minutes. He was unable to remember the event but was able to remember getting into the car and driving toward home. Clinical examination and an extensive investigation, including metabolic screening of blood (including random and fasting blood sugars, lipid profile, serum electrolyte, serum calcium, serum phosphate, serum magnesium), magnetic resonance imaging (MRI) of the brain, and routine EEG did not reveal a secondary cause for the presentation. He had been complaining of brief episodes of anterograde memory loss, which were attributed to age, anxiety, and stress during the previous 2 years before this accident. He also admitted to having a recent worsening of memory. Therefore, TEA was suspected over TGA, mainly owing to the history of recurrent episodes of memory loss and the fairly quick recovery of the amnestic episode. On suspicion, he was subjected to a sleep EEG to confirm a diagnosis of TEA, using Zeman’s criteria. The sleep EEG showed occasional bilateral sharpened theta transients suggesting a reduced seizure threshold. Together with the clinical context and the EEG findings, a probable diagnosis of TEA was made on the basis of Zeman’s criteria, and the patient was started on oxcarbazepine 200 mg twice a day. He was advised to stop driving. He did not have major incidents thereafter while on treatment; only three episodes of memory impairment of around 15 minutes were noted subsequently over the next 2 years, with the frequency becoming less and less. The amnestic episodes were mostly associated with sudden waking up from sleep and got better in about 1–2 hours of good sleep. However, no involuntary movements were noted; therefore, the patient and the family were not very convinced of the diagnosis of epilepsy. This led to reduced drug compliance, especially after about 1 year of treatment with antiepileptics. The family attributed memory impairment to aging and social issues. However, the patient was worried about his progressive memory loss, especially regarding the names of friends and day-to-day events, but claimed that his long-term memory was good. He presented 4 years from the first amnestic episode; that is, 2 years after the car accident, with three repeated generalized tonic–clonic seizures needing treatment with intravenous levetiracetam to control seizures. Investigations performed during this episode, including biochemistry and MRI, did not show a secondary cause for the repeated seizures, but postictal routine EEG showed spike and wave activity in the left temporal leads, confirming the initial diagnosis. The patient admitted poor compliance with the antiepileptic medicines prescribed to him. He was restarted on carbamazepine and is now seizure free 6 years following the first symptoms of the disease, and is very compliant with carbamazepine, knowing the consequences of poor compliance. However, the family noticed that his interictal memory impairment was persistent, if not deteriorating, and that he was more emotionally labile than he used to be 6 years ago.

## Discussion

This patient, with an unremarkable background history, had several episodes of transient, anterograde amnesia with preserved consciousness and language abilities before a formal diagnosis of TEA was made. The initial diagnosis was difficult and was missed, as the subtle episodes of memory impairment were not considered pathological and were thought to be age-related and psychological. A definite organic pathology was looked for when he presented with hard evidence of amnesia with preserved consciousness, which led to a car accident. Of the differential diagnoses, TGA and TEA were the most likely. Differentiation between TGA and TEA was difficult at the beginning, but TEA was suspected over TGA owing to a history of recurrent episodes of transient amnesia, especially when waking up from sleep, and because the duration of memory loss was shorter compared with that seen in TGA. EEG with sleep deprivation supported the diagnosis. Therefore, TEA was tentatively diagnosed on the basis of Zeman’s criteria: (1) a history of recurrent witnessed episodes of transient amnesia; (2) cognitive functions other than memory judged to be intact during typical episodes by a reliable witness; and (3) evidence for a diagnosis of epilepsy based on one or more defined characteristics [13]. The patient was started on antiepileptics, taking into consideration the risks and benefits. Antiepileptics reduced the frequency of amnestic episodes, favoring the tentative diagnosis. His later presentation with a generalized tonic–clonic (GTC) seizure while not being on antiepileptics, possibly focal seizures with secondary generalization, and the postictal EEG finding of spike and wave activity in the left temporal leads confirmed the diagnosis without any doubt, according to Zeman’s criteria.

TEA is often underdiagnosed. The danger is that the symptoms can easily pass by unrecognized until they result in nasty consequences, such as an accident or a full-blown generalized seizure. They would have been labeled as “functional” had some serious consequence not happened alongside the amnestic episodes. The car accident that happened 4 years ago would have been mistakenly interpreted as TGA, as there were no convincing signs of a seizure, and the history of episodic memory loss was not elicited in the past medical history. However, considering the impact of the amnestic episode, the temporary driving suspension was appropriate pending a definitive diagnosis. The decision to start antiepileptic medications, taking into consideration the risk of having a second episode, was ideal, but compliance with treatment was poor, as the patient and the family were less convinced about the diagnosis owing to a lack of typical features of a seizure in the presentation. However, the seizure frequency is reduced even with poor medication compliance, probably because the disease is usually controlled with low doses of antiepileptics. However, he later presented with a GTC seizure, with postictal EEG showing seizure activity in the temporal lobes; the likely explanation is that the temporal lobe seizure became secondarily generalized in the patient’s poorly controlled disease.

Our patient was an elderly man and had recurrent episodes of transient memory impairment as well as interictal memory impairment, as described in the previous case series [14]. However, he presented with a generalized tonic–clonic seizure, which is not very commonly seen in TEA [15].

TEA is a subset of temporal lobe epilepsy with late-onset limbic epilepsy of unknown cause, principally affecting middle- to old-age individuals, leading to recurrent, brief episodes of isolated memory impairment of epileptic cause while other cognitive functions remain intact [13]. It has a male predominance. A recent review of 115 cases observed the onset of amnesia at an average age of 62 years, giving rise to amnestic episodes at a frequency of around one per month, typically lasting 15–30 minutes and often occurring on waking [15]. TEA is reported to be associated with interictal memory disturbances, including accelerated long-term forgetting, remote memory impairment and autobiographical and topographical memory impairment [1, 13]. Transient amnesia is the sole presentation in about 34% of patients and could be anterograde or retrograde. In addition, individuals may develop complex partial seizures typical of mesial temporal lobe epilepsy in two-thirds of the patients in whom olfactory hallucinations and features of automatism, such as chewing or lip-smacking movements, can be seen. However, secondarily generalized tonic–clonic seizures are rare. Emotional lability has been reported in 40% of patients. Only 36% of patients showed epileptiform discharges localized to temporal lobes during interictal routine EEG [15]. Prolonged, sleep-deprived, or ambulatory EEG could complement diagnosis when routine EEGs are normal. Epileptiform abnormalities mainly involve the frontal and/or temporal regions. MRI is unremarkable in the majority of patients. However, MRI may show hippocampal atrophy or focal structural lesions in the temporal lobes. Fluorodeoxyglucose (FDG)-positron emission tomography (PET) together with an MRI of the brain help to distinguish TEA from neurodegenerative disease, when suspected. TEA is treatable and the seizures are usually controlled with a single, low dose of a usual antiepileptic such as carbamazepine, lamotrigine, or sodium valproate [16]. The seizures respond promptly to treatment, but the interictal memory loss tends to persist [1]. More than 90% of patients reported complete cessation of seizures following the initiation of antiepileptic medications. In total, 59 out of 96 patients with TEA experienced complete seizure cessation in a case series [15]. The optimal duration of anticonvulsant treatment is not reported and will need to be clinically decided. TEA is associated with “atypical” forms of memory disturbances, including accelerated long-term forgetting, disproportionate autobiographical amnesia, and topographical amnesia [15]. However, an increased risk of dementia has not been seen in a case series of ten patients followed-up for 20 years, and the prognosis appeared generally benign [14]. Moreover, life expectancy is not reduced in TEA [17]. The reason for the onset of the disease in middle/old age is not fully understood, and no particular connection with vascular risk factors is noted [17]. However, age-related susceptibility and male predominance point to the possible relevance of hormonal factors. Encephalitis caused by antibodies to N-methyl-D-aspartate (NMDA) receptors has not previously been documented in TEA, however, there is one case report of a man in his 40s presenting with TEA, with high NMDA receptor levels [18]. Another recent study published in 2023 evaluating cerebrospinal fluid (CSF) of 125 patients with TEA found a subset of patients with red flags of degenerative diseases to have amyloid and tau biomarkers, indicating that TEA may be the starting presentation of some cases of Alzheimer’s disease [19].

Clinical differentiation of causes for transient amnesia are presented in Table 1. The most characteristic features of TEA over others are brevity and recurrence of episodes [13]. TGA is characterized by a sudden onset of dramatic anterograde amnesia lasting up to 24 hours, and this usually does not recur [20]. Transient amnesia is a very uncommon presentation of a TIA. Further, TIAs are common and usually do not recur, unlike TEA [12]. Functional amnesias usually have preceding psychological stress or trauma and the presence of loss of personal identity as a main finding. They could have variable pathologically unexplainable symptoms [21, 22]. Differentiation between causes is important, as treatment and prognosis depend on it. TEA is treated with antiepileptics and can be associated with long-term memory impairment, while there is no proven treatment for TGA nor is it associated with memory impairment. TIAs need good risk factor control to prevent future cerebrovascular events. A recent study has proposed a new scoring system, the EPIlepsy AMNEsia (EPIAMNE) score, using quantitative EEG analysis as a tool for differentiating TEA from TGA. They observed the EPIAMNE score as being able to detect TEA with higher accuracy than using standard EEG and symptoms only (23).

**Table 1:** Differentiating features of different causes of transient amnesia

	TEA	TGA	TIA	Functional
Typical age	50 years	50 years	50 years	Any age
Duration	< 1 hour	4–6 hours	Variable	Days–months
Amnesia	Mix of anterograde and retrograde	Dense anterograde	Not well characterized	Mainly retrograde
Other features	Olfactory hallucinations, automatism		Focal neurological signs	Mood disturbance
Precipitants	On waking up from sleep	Physical or psychological stresses		Stressful life events
Recurrence	Common	Rare (6–10%)	Not characterized	May recur if psychological fatigue recurs
Risk factors and past medical history	Not characterized	Migraine	Cerebrovascular risk factors	Psychiatric illness or substance abuse
Interictal or postictal memory impairment	Present and may progress	No permanent deficit	Risk of permanent deficit following completed stroke	Variable

*TEA* transient epileptic amnesia, *TGA* transient global amnesia, *TIA* transient ischemic attack

This case highlights several important points in the diagnosis and management of TEA. A careful history of the presentation, especially regarding recurrent episodes, is important to differentiate between TEA and TGA. Transient memory loss in older adults, especially on sudden waking up from sleep, along with memory decline, is the classic presentation of TEA. A high degree of suspicion of TEA on first presentation and follow-up of these patients for progression and recurrence of symptoms is important in evaluating a patient presenting with transient memory loss for the first time in life, as interictal EEG could be normal, and early use of prolonged EEG with emphasis on sleep monitoring could aid in making the diagnosis. Temporary suspension of driving in patients presenting with transient amnesia is best practice until the diagnosis is confirmed. Similar to treating any other epilepsy, the clinician may decide to start an antiepileptic medication if risks of having a fit outweigh the risk of treatment. Moreover, educating the patient about the tentative diagnosis, the usual course of the disease, and explaining the plan to review medications, depending on the disease progression, will help improve medication compliance in patients.

## Conclusion

TEA is a treatable cause of transient amnesia in older people, often mistaken for senile dementia, cerebrovascular disease, or functional amnesia. Reports on the long-term outcomes of partially treated TEA are limited. We report temporal progression of partial TEA over 6 years, culminating in generalized tonic–clonic seizures. This case shows that the diagnosis of TEA is difficult on first presentation and can be mistaken for age-related memory loss or psychological conditions. However, a high degree of suspicion of TEA as a differential diagnosis is important. A history of recurrent, brief amnestic episodes and interictal memory impairment helps to suspect the diagnosis, even with a normal interictal EEG. An active diagnosis of TEA is very important, as it is an easily treatable condition, and without timely treatment could give rise to dangerous consequences and even death, following accidents.

## Data Availability

Not applicable.

## References

[1] Zeman A, Butler C. Transient epileptic amnesia. Curr Opin Neurol. 2010;23(6):610–6. 20885322 10.1097/WCO.0b013e32834027db

[2] Butterworth RF. 6 edn. Siegel GJ AB, Albers RW, *et al*. editors. Philadelphia: Lippincott-Raven; 1999.

[3] Ghosh A. Endocrine, metabolic, nutritional, and toxic disorders leading to dementia. Ann Indian Acad Neurol. 2010;13(Suppl 2):S63–8. 21369420 10.4103/0972-2327.74247 PMC3039161

[4] Tong DC, Grossman M. What causes transient global amnesia? New Insights DWI. 2004;62(12):2154–5. 10.1212/01.wnl.0000132256.64800.cb 15210872

[5] Owen D, Paranandi B, Sivakumar R, Seevaratnam M. Classical diseases revisited: transient global amnesia. Postgrad Med J. 2007;83(978):236–9. 17403949 10.1136/pgmj.2006.052472 PMC2600033

[6] Asadi-Pooya AA. Transient epileptic amnesia: a concise review. Epilepsy Behav. 2014;31:243–5. 24230990 10.1016/j.yebeh.2013.10.021

[7] Cho S, Lee WW, Kang K, Park JM, Kim BK, Kwon O, *et al*. Transient epileptic amnesia with preserved consciousness: a report of two cases. J Epilepsy Res. 2017;7(1):54–6. 28775957 10.14581/jer.17010 PMC5540692

[8] Ramanan VK, Morris KA, Graff-Radford J, Jones DT, Burkholder DB, Britton JW, *et al*. Transient epileptic amnesia: a treatable cause of spells associated with persistent cognitive symptoms. Front Neurol. 2019. 10.3389/fneur.2019.00939. 31555199 10.3389/fneur.2019.00939 PMC6724577

[9] Easton JD, Saver JL, Albers GW, Alberts MJ, Chaturvedi S, Feldmann E, *et al*. Definition and evaluation of transient ischemic attack: a scientific statement for healthcare professionals from the American Heart Association/American Stroke Association Stroke Council; Council on Cardiovascular Surgery and Anesthesia; Council on Cardiovascular Radiology and Intervention; Council on Cardiovascular Nursing; and the Interdisciplinary Council on Peripheral Vascular Disease. The American Academy of Neurology affirms the value of this statement as an educational tool for neurologists. Stroke. 2009;40(6):2276–93. 19423857 10.1161/STROKEAHA.108.192218

[10] Nadarajan V, Perry RJ, Johnson J, Werring DJ. Transient ischaemic attacks: mimics and chameleons. Pract Neurol. 2014;14(1):23–31. 24453269 10.1136/practneurol-2013-000782 PMC3913122

[11] Zorzon M, Antonutti L, Masè G, Biasutti E, Vitrani B, Cazzato G. Transient global amnesia and transient ischemic attack. Stroke. 1995;26(9):1536–42. 7660394 10.1161/01.str.26.9.1536

[12] Michel P, Beaud V, Eskandari A, Maeder P, Demonet J-F, Eskioglou E. Ischemic amnesia. Stroke. 2017;48(8):2270–3. 28584000 10.1161/STROKEAHA.117.017420

[13] Zeman AZ, Boniface SJ, Hodges JR. Transient epileptic amnesia: a description of the clinical and neuropsychological features in 10 cases and a review of the literature. J Neurol Neurosurg Psychiatry. 1998;64(4):435–43. 9576532 10.1136/jnnp.64.4.435 PMC2170058

[14] Savage SA, Butler CR, Hodges JR, Zeman AZ. Transient epileptic amnesia over twenty years: long-term follow-up of a case series with three detailed reports. Seizure. 2016;43:48–55. 27886629 10.1016/j.seizure.2016.10.022 PMC6161809

[15] Baker J, Savage S, Milton F, Butler C, Kapur N, Hodges J, *et al*. The syndrome of transient epileptic amnesia: a combined series of 115 cases and literature review. Brain Commun. 2021;3(2):fca038. 10.1093/braincomms/fcab038 PMC8047097 33884371

[16] Ramanan VK, Morris KA, Graff-Radford J, Jones DT, Burkholder DB, Britton JW, *et al*. Transient epileptic amnesia: a treatable cause of spells associated with persistent cognitive symptoms. Front Neurol. 2019;10:939. 31555199 10.3389/fneur.2019.00939 PMC6724577

[17] Savage SA, Baker J, Milton F, Butler C, Zeman A. Clinical outcomes in transient epileptic amnesia: a 10-year follow-up cohort study of 47 cases. Epilepsia. 2022;63(5):1115–29. 35253220 10.1111/epi.17214 PMC9310913

[18] Savage SA, Irani SR, Leite MI, Zeman AZ. NMDA receptor antibody encephalitis presenting as transient epileptic amnesia. J Neuroimmunol. 2019;327:41–3. 30686545 10.1016/j.jneuroim.2019.01.011 PMC6367595

[19] Cretin B, Philippi N, Bousiges O, Blanc F. Transient epileptic amnesia: a retrospective cohort study of 127 cases, including CSF amyloid and tau features. J Neurol. 2023;270(4):2256–70. 36715748 10.1007/s00415-023-11576-7

[20] Bartsch T, Deuschl G. Transient global amnesia: functional anatomy and clinical implications. Lancet Neurol. 2010;9(2):205–14. 20129169 10.1016/S1474-4422(09)70344-8

[21] Kritchevsky M, Chang J, Squire LR. Functional amnesia: clinical description and neuropsychological profile of 10 cases. Learn Mem. 2004;11(2):213–26. 15054137 10.1101/lm.71404 PMC379692

[22] Reus VI. Chapter 52—functional neurologic symptom disorders. In: Aminoff MJ, Josephson SA, editors. Aminoff’s neurology and general medicine. 5th ed. Boston: Academic Press; 2014. p. 1069–85.

[23] Sancetta BM, Ricci L, Assenza G, Boscarino M, Narducci F, Vico C, *et al*. EPIAMNE: a new scoring system for differentiating transient EPIleptic AMNEsia from transient global amnesia. Brain Sci. 2022. 10.3390/brainsci12121632. 36552092 10.3390/brainsci12121632 PMC9775429

